# Intracardiac tumor as a rare manifestation of genetic syndromes—presentation of a family with Gorlin syndrome and a literature review

**DOI:** 10.1007/s13353-020-00582-4

**Published:** 2020-09-22

**Authors:** Krzysztof Szczałuba, Ewa Makuła, Anna Piórecka-Makuła, Justyna Sicińska, Małgorzata Rydzanicz, Piotr Gasperowicz, Rafał Płoski, Bożena Werner

**Affiliations:** 1 Department of Medical Genetics, Medical University of Warsaw, ul Pawinskiego 3c, 02-106 Warsaw, Poland; 2 Medical University of Warsaw, Warsaw, Poland; 3 Department of Pediatric Cardiology and General Pediatrics, Medical University of Warsaw, Warsaw, Poland; 4 Clinical Department of Dermatology, Central Clinical Hospital of the MSWiA, Warsaw, Poland

**Keywords:** Cardiac tumor, Fibroma, Gorlin syndrome, Familial, Exome sequencing

## Abstract

Intracardiac tumors in children are relatively rare, but their clinical consequences may include severe outflow tract obstruction, embolism, cardiac insufficiency, or rhythm disturbances. In some cases, the tumor may constitute part of a genetic condition and prompt additional investigations, as well as a modification of therapeutic management. Herein, we present a molecularly confirmed familial case of Gorlin syndrome with an early cardiac tumor as a presenting sign. We provide detailed clinical characteristics of the affected individuals and a useful review of syndromic causes of pediatric cardiac tumors in clinical practice.

## Introduction

Cardiac tumors are benign or malignant neoplasms that may originate in all the three layers of heart tissue (Uzun et al. 2007). Their frequency in fetal life is estimated at 0.14%, while postnatal population prevalence reaches 0.5% in cases of children evaluated for cardiac symptoms (Uzun et al. 2007; Beghetti et al. 1997; Linnemeier et al. 2015). About 90% of the primary cardiac neoplasms have benign characteristics. The most common type is rhabdomyoma (up to 60%) followed by teratoma, fibroma, and myxoma (Holley et al. 1995, Uzun et al. 2007; Tzani et al. 2017). In the majority of patients, intracardiac tumors are isolated findings on fetal or infantile echocardiogram, while in the rest, they are part of a genetic syndrome diagnosis (Vidaillet 1988).

Robert Gorlin estimated that in his series, close to 4% of all cardiac fibromas were part of nevoid basal cell carcinoma syndrome (NBCCS, Gorlin syndrome) (OMIM:109400) (Gorlin 1987). NBCCS is characterized by lamellar or early calcification of the falx, jaw keratocysts, palmar and/or plantar pits, and multiple and/or early-onset basal cell carcinomas (Gorlin 2004; Bree et al. 2011). Early diagnosis of the syndrome enables optimal personalized care. Majority of Gorlin syndrome cases are caused by mutations in *PTCH1* (*Patched1*) gene, which is part of the Hedgehog (Hh) molecular pathway (Johnson et al. 1996).

Herein, we show a molecularly confirmed familial case of Gorlin syndrome consisting of an infant proband presenting with intracardiac tumor, polydactyly, and macrocephaly and the father with features of macrocephaly, mitten-type syndactyly of fingers, polydactyly, and maxillary cysts. We describe current and future targeted treatment options in our familial case and provide a review of syndromic causes of pediatric intracardiac tumors.

## Family report

We present a case of an infant girl with multiple dysmorphic features and a cardiac tumor of unknown origin diagnosed postnatally. The girl was born preterm at 36 weeks’ gestational age with the birth weight of 4000 g by caesarean section due to maternal hypertension. The Apgar score was 3 and 5 at 1 and 5 min after birth, respectively. After initial resuscitation, the transthoracic echocardiography was performed revealing a tumor in the left ventricle. The neonate was referred to the department of pediatric cardiology for subsequent diagnostics.

**Fig. 1:**
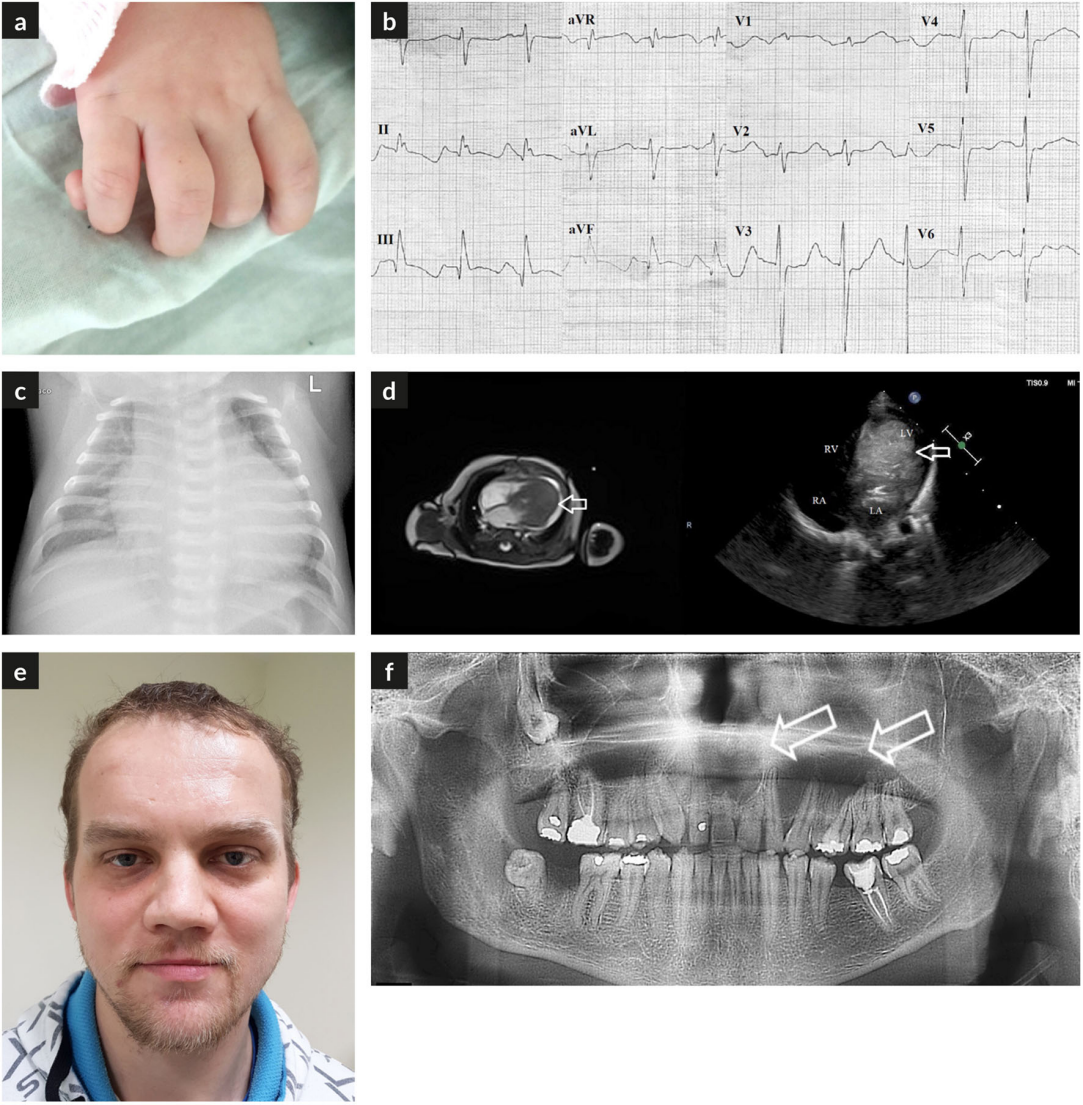
**a** An additional phalanx of fifth finger in the proband. **b** The 12-lead electrocardiogram of the proband demonstrating sinus rhythm with delayed intraventricular conduction and generalized repolarization abnormalities. **c** The anteroposterior projection chest radiograph demonstrating cardiomegaly. **d** The transthoracic echocardiography, apical four-chamber view demonstrating a large bean-shaped tumor of the left ventricle. **e** Macrocephaly and frontal bossing in the father. **f** Pantomogram of the father showing maxillary cysts

On admission, the child was in a serious but stable condition with normal resting heart rate of 150 beats per minute (bpm) and arterial blood pressure of 81/68 mmHg. Blood oxygen saturation was 92–94%, and capillary refill time was normal. Macrocephaly with head circumference of 38.5 cm (> 97th centile), bilateral polydactyly of fingers with unilateral polydactyly of toes, and a natal tooth on lower gingiva were observed (Fig. 1a). Dyspnea signs were present with retractions of lower intercostal spaces and tachypnea (70/min). Heart auscultation revealed systolic murmur graded 2/6 on Levine scale heard over the whole heart. The liver edge was palpable 1.5 cm below the costal margin. The remaining physical examination was unremarkable.

Laboratory testing revealed anaemias (hemoglobin concentration of 10.8 g/dL) and significantly increased NT-proBNP level (26,500 pg/ml) (the upper norm for NT-proBNP at 598 pg/ml was established by Lin et al. 2013). Tumor lysis laboratory markers (uric acid, potassium, lactate dehydrogenase) were within the normal limits. The 12-lead electrocardiogram (ECG) showed sinus rhythm with delayed intraventricular conduction and generalized repolarization abnormalities—inverted T waves in leads II, III, aVF, V4-V6, and low-amplitude T waves in leads I and aVL (Fig. 1b). The anteroposterior chest radiograph was suggestive of cardiomegaly with cardiothoracic ratio of around 0.7 (Fig. 1c).

Two-dimensional transthoracic echocardiography revealed a large bean-shaped tumor (size, 50 × 30 mm) attached to the posterolateral wall of left ventricle with the areas of hyper- and hypodensity (Fig. 1d). Moderate pericardial effusion with late diastolic compression of the right atrium was found. Patent ductus arteriosus (PDA) with diameter of 3.5 mm with left-to-right shunt coexisted with dilation of the pulmonary trunk and pulmonary arteries.

Cardiac magnetic resonance confirmed echocardiographical findings and revealed possible diaphragm and parietal pericardium invasion. Contractility of left ventricle wall was impaired by presence of the tumor mass. Peripheral contrast enhancement suggested the presence of tumor capsule, and the lack of central enhancement suggested necrosis. Additionally, oval mass (size, 16 × 10 mm) localized between the anterior and lateral wall of left ventricle with peripheral contrast enhancement similar to the main tumor was described; however, the connection between them could not be excluded. Imaging studies suggested rhabdomyoma, fibroma, or sarcoma.

Subsequent treatment included administration of spironolactone, furosemide, and captopril. Hemodynamically significant PDA was closed with PDA occluder. Signs of dyspnea decreased. Because of the high risk of thromboembolic events, cardiac tamponade, arrhythmia, and possible presence of metastases, the biopsy during the procedure was not performed.

Magnetic resonance imaging of central nervous system showed no tumors. Rhabdomyoma associated with tuberous sclerosis could still not be excluded; thus, therapy with sirolimus was started. After 4 weeks, no significant changes in tumor size were observed, and sirolimus therapy was discontinued. Pericardial drainage due to fluid accumulation was performed. Subsequent specimen cytology was negative for tumor cells.

Additionally, wide QRS extra beats were observed during hospitalization in the standard ECG monitoring. Twenty-four-hour Holter monitoring revealed a few episodes of ventricular tachycardia up to 200 bpm. Metoprolol was administered, and during subsequent tests, only single premature ventricular beats were observed. Pending genetic test results the infant was discharged in a good general condition.

The 29-year-old father of the proband girl presented with macrocephaly (head circumference 61 cm, > 97th centile) and frontal bossing, mitten-hand–type bilateral syndactyly, and unilateral postaxial hand polydactyly (Fig. 1e). He had a history of maxillary cyst surgery at age 22 (Fig. 1f). He had no other medical issues and otherwise was normal physically and intellectually.

## Genomic testing

In the proband, exome sequencing (ES) was performed using *Nextera Flex for Enrichment* sample preparation kit combined with TruSeq DNA Exome (45 Mb) probes (Illumina, San Diego, CA, USA) according to the manufacturer’s instruction. Library was pair-end sequenced (2× 100 bp) on HiSeq 1500 (Illumina) to the mean depth 83×, 98.7% of target was covered ≥ 10×, and 94.4% was covered ≥ 20×. Bioinformatics analysis of raw ES data and variant prioritization were performed as previously described (Rydzanicz et al. 2019). Selected variants were further validated in the proband, her parents, and grandparents (father’s parents) by amplicon deep sequencing (ADS) performed using Nextera XT Kit (Illumina) and sequenced on HiSeq 1500 (Illumina).

**Fig. 2:**
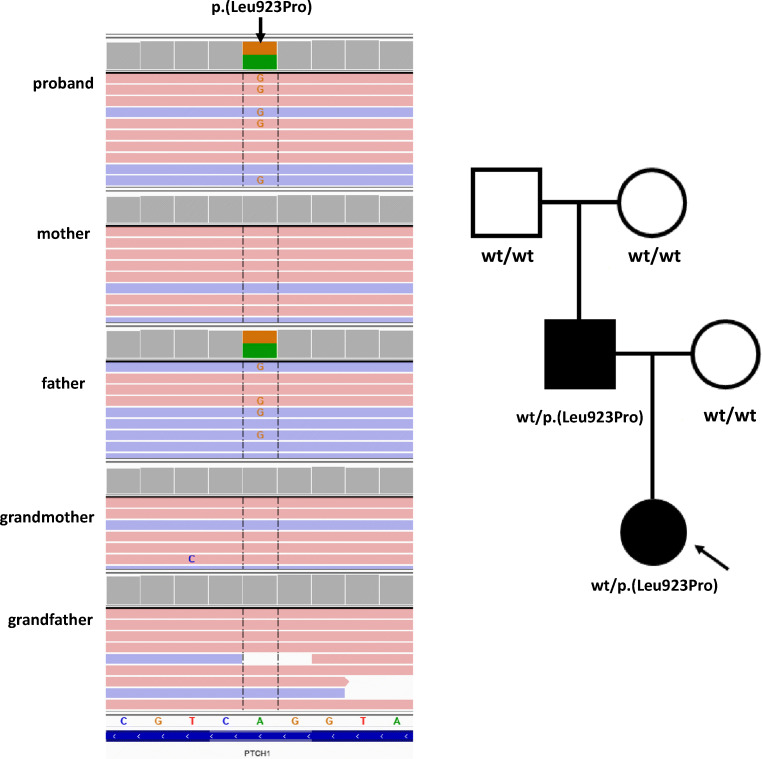
Results of ES analysis in the family

Considering the patient’s phenotype and the characteristics of the variants, we prioritized four ultrarare variants in *ASH1L*, *CDKN2A*, *SIX4*, and *PTCH1* genes for verification and family study. Variants in *ASH1L*, *CDKN2A*, and *SIX4* were inherited from the healthy mother, while the variant in the *PTCH1* gene (hg38; g.chr9:095459719-A>G, NM_000264.5:c.2768T>C, p.(Leu923Pro)) was inherited from the affected father. The p.(Leu923Pro) variant in PTCH1 was absent from the proband’s grandparents’ blood. Therefore, we concluded that this variant occurred de novo in the proband’s father and was transmitted to the proband in an autosomal dominant manner of inheritance (Fig. 2). The p.(Leu923Pro) was absent in all queried databases, including in-house database of > 3000 ES of Polish individuals, and was predicted to be damaging by in silico predictors using by Varsome (Kopanos et al. 2019).

## Discussion

Neonatal cardiac tumors are predominantly rhabdomyomas. Multiple tumors suggest the diagnosis of tuberous sclerosis while in case of single lesions, fibromas, myxomas, pericardial teratomas, and hemangiomas should be taken into account. Isolated cases constitute the majority of pediatric presentations of intracardiac tumors. In such individuals, both physical and intellectual developments remain unaffected. The management is restricted either to surgery in cases of ventricular outflow tract obstruction, embolism, and pulmonary/cardiac insufficiency or, most commonly, to monitoring cardiac function for a likely regression of the usually benign tumor. As the tumor shrinks in size, the foci of rest cells may create a background for rhythm disturbances (Mankad and Herrmann 2016). In the usually benign and slow-growing intracardiac tumors, sometimes, the parents have to be screened for familial occurrences, e.g., familial myxoma syndrome. While rare, the genetic syndromes featuring intracardiac tumors present bigger challenges, both in terms of diagnosis and treatment. Our literature search on genetic conditions with intracardiac tumors revealed 10 fetal and pediatric entities that are shown in detail in Table 1. Apparently, there are distinct clinical features that prove helpful in establishing an early diagnosis of a certain genetic condition. Multiple tumors suggest an underlying tuberous sclerosis, especially in the setting of skin, kidney, and CNS typical findings. Cardiac tumors are also present as single occurrences in two other phakomatoses: Gorlin (NBCCS) syndrome and neurofibromatosis type 1 (NF1). In some conditions, establishing the pathological type of tumor may facilitate diagnosis. Myxoma at an early age, often multicentric and affecting any cardiac chamber, may imply the diagnosis of Carney complex in up to 23–32% of affected individuals (Vidaillet 1988; Bertherat et al. 2009). Cardiac lipoma suggests Cowden syndrome, while neurofibroma and paraganglioma point to NF1 and possible hereditary paraganglioma/pheochromocytoma syndromes, respectively. A critical implication of a syndromic intracardiac tumor diagnosis is a necessity for early-onset screening for extracardiac neoplasms in affected individuals. In 9 out of 10 conditions listed in Table 1, malignancies may arise as part of their clinical picture. Especially stringent guidelines for tumor screening have been established in Beckwith-Wiedemann syndrome characterized by bodily and internal organ overgrowth as well as embryonal tumors of liver and kidneys.

**Table 1:** Differential diagnosis of syndromic presentations of fetal and pediatric primary intracardiac tumors

Genetic syndrome	Clinical characteristics	Cardiac features	Inheritance	Gene/locus	OMIM	Literature
Tuberous sclerosis (TS)	Abnormalities of the skin (hypomelanotic macules, confetti skin lesions, facial angiofibromas, shagreen patches, fibrous cephalic plaques, ungual fibromas); brain (subependymal nodules, cortical dysplasias, and subependymal giant cell astrocytomas, seizures, intellectual disability/developmental delay, psychiatric illness); kidney (angiomyolipomas, cysts, renal cell carcinomas); heart (rhabdomyomas, arrhythmias); and lungs (lymphangioleiomyomatosis, multifocal micronodular pneumonocyte hyperplasia)	Rhabdomyomas, (especially if more than one) in up to 50% of confirmed cases	Autosomal dominant	*TSC1/TSC2*	191100/613254	Sancak et al. (2005)
Gorlin syndrome (nevoid basal cell carcinoma syndrome) (NBCCS)	Multiple nevoid, cystic, pigmented or keratotic basalomas; Multiple jaw cysts; cystic alterations of long bones; costal anomalies, kyphoscoliosis, occult spina bifida, funnel chest; agenesis of corpus callosum, calcification of falx cerebri; edged skull, widened nasal bridge, hypertelorism; palmar and plantar keratosis; ovarian fibromas, male hypogonadism, cryptorchidism	Fibroma	Autosomal dominant	*PTCH1*, *PTCH2*, *SUFU*	109400	Bossert et al. (2006) Ritter et al. (2018) Watson et al. (2004) Doede et al. (2004) Foulkes et al. (2017)
Carney complex type 1 (CNC1)	Cardiac, endocrine, cutaneous, and neural myxomatous tumors; variety of pigmented lesions of the skin and mucosae	Myxoma at a young age (may be multicentric and affect any heart chamber)	Autosomal dominant	*PRKAR1A*	160980	Stratakis and Raygada (2003) Bertherat et al. (2009)
Carney complex variant?	Trismus, pseudocamptodactyly and freckling	Myxoma (familial?)	Autosomal dominant?	*MYH8?*	608837	Veugelers et al. (2004) Stratakis et al. (2004)
Beckwith-Wiedemann (BWS)	Overgrowth, macroglossia, omphalocele, hemihypertrophy	Rhabdomyoma or angiofibroma or hamartoma in the setting of macrosomia	Imprinting disorder	11p15 region (loci IC1 and IC2; *CDKN1C*, *KCNQ10T1*, *KCNQ1*, *IGF2*, *H19* genes)	130650	Longardt et al. (2014) Reddy et al. (1972) Satgé et al. (2005)
Neurofibromatosis type 1	Characteristic skin changes, neurofibromas, optic gliomas	Neurofibroma	Autosomal dominant	17q11.2micro-/deletions involving *NF1*	613675	Nguyen et al. (2013)
Birt-Hogg-Dube (BHD)	Lung cysts, colonic polyps, renal tumors	Rhabdomyoma	Autosomal dominant	*FLCN*	135150	Toro et al. (2008)
Hereditary paraganglioma/pheochromocytoma syndromes (including Carney-Stratakis syndrome and Carney triad)	Paragangliomas, gastric stromal sarcomas or leiomyosarcomas, pulmonary chondromas	Paraganglioma	Autosomal dominant?	*SDHB*, *SDHC*, *SDHD*	606864, 604287	Miraldi et al. (2007)
Cowden syndrome (CS)	Macrocephaly, hamartomas, ovarian cysts, cancer	Lipoma	Autosomal dominant	*PTEN*	158350	Ceresa et al. (2010)
Familial myxoma	Atrial myxomas	Myxoma	Autosomal dominant	*PRKAR1A*	255960	Singh and Lansing (1996)

In our familial case, the final diagnosis was established by ES but combined clinical exam of the proband, and the father enabled inclusion of Gorlin syndrome in the differential diagnosis. The key symptoms present in the father were history of jaw cysts, macrocephaly, syndactyly, and polydactyly. Thus, this case best resembles Doede et al.’s description of an infant boy with macrocephaly and finger syndactyly and his mother with macrocephaly and facial fibromas (Doede et al. 2004). According to the authors, combined syndactyly of fingers and toes coupled with a cardiac tumor strongly suggests syndromic diagnoses. Indeed, the father of our proband had a complete syndactyly of the hands as well as unilateral polydactyly, and cardiac tumor with polydactyly was observed in the proband. Familial occurrence of Gorlin syndrome and the value of examining the probands’ parents were underlined by other authors. Ritter et al. reported jaw cysts in the father of an affected girl, and in Bossert et al.’s case, the mother of the proband had already been diagnosed with NBCCS (Ritter et al. 2018; Bossert et al. 2006).

In the presented infant girl, a therapeutic trial of sirolimus was initiated. This is based on an assumption that tuberous sclerosis (TS) is the most frequent syndromic presentation of cardiac tumors, and mTOR inhibitors are effective in treating CNS and kidney neoplasms of TS (Curatolo and Moavero 2012). Moreover, it may be difficult to exclude this condition in an early infantile period. However, the treatment was discontinued due to lack of effect. Unsurprisingly, the majority of tumor pathologies seen in NBCCS are fibromas, and there is no evidence of disrupted mTOR signaling in these tumors. Instead, loss of *PTCH1* locus has been shown in cardiac fibroma tissue (Scanlan et al. 2008). Recently, in a mixed fibrous/myxoid type of gastric neoplasm called plexiform fibromyxoma, a link was established between Hedgehog signaling (Hh) and loss of *PTCH1* (Banerjee et al. 2019). Treatment of the primary tumor cells with the Hh pathway inhibitor, sonidegib (LDE225, Novartis), resulted in dose-dependent cell killing (Banerjee et al. 2019). This provides rationale for the future therapeutic trials of other tumors in the setting of *PTCH1* loss, and it would be interesting to see how various types of tumors in Gorlin syndrome respond to the similar treatment.

In the described proband and her affected father, we have identified through ES a missense variant NM_000264.4:c.2768T>C in the *PTCH1* gene creating a substitution p.(Leu923Pro). According to Varsome, most missense changes in *PTCH1* are of uncertain significance. However, this particular variant is absent from GnomAD or our in-house databases of variants, and in silico analyses are predictive of its pathogenicity. Moreover, the variant was excluded in healthy paternal grandparents of our proband. Thus far, somatic mutation p.Leu923Pro has only been found in childhood medulloblastomas as the Sonic Hedgehog (SHH) pathway is likely involved in progression of these tumors (Parsons et al. 2011; Iorgulescu et al. 2018). Medulloblastomas are part of the Gorlin syndrome where they may appear at ages 1–2 years, which is much earlier than expected in sporadic cases (Cowan et al. 1997). Thus, it may be reasonable to provide MRI brain imaging in such instances.

In summary, we present a familial case of Gorlin syndrome characterized by the presence of a rare intracardiac tumor in the proband. Clearly, the syndromic diagnosis enabled optimization of therapeutic management, and it may support introduction of specific prophylactic measures. Similar personalized management should be introduced once the diagnosis of any of the genetic syndromes presenting with primary intracardiac tumors and discussed here is reached.
